# Radical Operation for the Cure of Incipient Hip-Joint Disease—Report of Case1Read at the meeting of the Medical Society of the County of Erie, April 20, 1908

**Published:** 1909-06

**Authors:** H. C. Rooth

**Affiliations:** Clinical Instructor in Surgery, University of Buffalo, Surgeon Riverside Hospital


### Radical Operation for the Cure of Incipient Hip-joint Disease—Report of Case.[1]

By H. C. ROOTH, M. D.
Clinical Instructor in Surgery, University of Buffalo, Surgeon Riverside Hospital.

AS tubercular coxitis is comparatively common in children, especially among the poorer classes and in tenement districts, any method that will hasten recovery, without incurring too great a risk, should receive our serious attention.

The usual treatment employed requires from one to two or three years to effect a cure, and even then deformity or perhaps ankylosis of the hip may remain.

While the treatment in most common use, such as tonics, weight and pulley, splints, plaster dressings, crutches, and the like, yields excellent results in many cases, if intelligently employed, yet the time required and the expense attached to so long a period of treatment become a great burden.

If the diseased focus or foci can be removed by operation, we can spare our patients many months of suffering and invalidism, and I am sure that in selected cases this can be done.

Five years ago Dr. Herman Mynter returned from Copenhagen enthusiastic over the results which were being obtained there in cases of incipient hip-joint disease, which were treated by trephining the neck of the femur through the trochanter major and thereby removing the diseased area.

This method was quite popular some years ago, but owing to the fact that the disease is not always confined to the neck, or even to the epiphysis, the affected areas were not always removed, and consequently the results were at times disappointing. Since the introduction of the *x*-ray, however, this objection, it seems to me, has lost its weight, for we can determine quite accurately the seat or location of the disease, and therefore can select our cases intelligently.

I cannot see why a tuberculous focus located in the neck of the femur should not be removed with the same promptness as we remove the same disease if located in other accessible parts of the body.

The case I wish to report is as follows:

T. S., female, age 6 years, first seen by me September 1, 1903. Early in the spring of this year she began to complain of pain in her right hip and showed a slight limp. There was some spasm of muscles and other symptoms of hip-joint trouble. She was seen at this time by Dr. J. C. Thompson, who prescribed tonics and rest and had her taken to the country. Under this treatment

---

1. Read at the meeting of the Medical Society of the County of Erie, April 20, 1908

she improved and all of her symptoms disappeared. She remained apparently cured until two weeks before I saw her (four months) when the doctor was again consulted on account of a return of her former symptoms, only in a much more severe and acute form. He then made an *x*-ray examination and located a spot in the neck of the femur, inner third, which showed marked signs of softening.

At this time she was referred to me, and I advised radical operation for the removal of the diseased focus. She then consulted one of our leading orthopedic surgeons, who advised against operation and had her removed to a hospital and placed in bed with weight and pulley. She remained there two days and then returned to me for operation. On September 4, 1903, assisted by Dr. Thompson, I exposed the hip-joint and trephined through the neck of the femur from the great trochanter to the head, but not through it. The size of the trephine was one-half inch. Then with a sharp spoon this canal was curetted and the instrument soon broke through the neck at the point of disease, as indicated by the *x*-ray. This was soft and easily removed, leaving a hole about three-quarters of an inch in diameter. The joint was then thoroughly washed and the capsule sewed with catgut sutures, and the wound closed without drainage. A plaster-of-paris splint was applied and she was placed in bed with slight traction on the limb. This splint was removed in two weeks, at which time the wound was entirely healed. Another splint was applied and allowed to remain two weeks more, the slight traction being continued. At this time the leg could be handled and fully flexed on the body without pain, and all spasm of muscles had disappeared. By October 24, or seven weeks after the operation, she was up and using the leg for two hours each day, and in ten weeks was well. At this time, through the courtesy of Dr. Park, I presented the patient at a meeting of the American Surgical Society. It is now five years since the operation and she has remained in perfect health since that time.

It would seem from the result obtained in this case that this form of treatment deserves more attention than it has been receiving in the last few years.

350 ASHLAND AVENUE.

